# Conductive Atomic Force Microscopy Study of the Resistive Switching in Yttria-Stabilized Zirconia Films with Au Nanoparticles

**DOI:** 10.1155/2018/5489596

**Published:** 2018-07-02

**Authors:** Dmitry Filatov, Inga Kazantseva, Dmitry Antonov, Ivan Antonov, Maria Shenina, Dmitry Pavlov, Oleg Gorshkov

**Affiliations:** ^1^Research and Educational Center for Physics of Solid State Nanostructures, Lobachevsky State University of Nizhny Novgorod, 23 Gagarin Ave., Nizhny Novgorod 603950, Russia; ^2^Department of Physics, Lobachevsky State University of Nizhny Novgorod, 23 Gagarin Ave., Nizhny Novgorod 603950, Russia; ^3^Research Institute for Physics and Technology, Lobachevsky State University of Nizhny Novgorod, 23 Gagarin Ave., Nizhny Novgorod 603950, Russia

## Abstract

We report on the investigation of the resistive switching (RS) in the ultrathin (≈5 nm in thickness) yttria-stabilized zirconia (YSZ) films with single layers of Au nanoparticles (NPs) by conductive atomic force microscopy (CAFM). Besides the butterfly-type hysteresis loops in the current-voltage (*I*-*V*) curves of the contact of the CAFM probe to the investigated film surface corresponding to the bipolar RS, the negative differential resistance (NDR) has been observed in the *I*-*V* curves of the AFM probe contact to the YSZ films with Au NPs in the conductive (“ON”) state. The NDR has been related to the resonant tunneling of electrons through the size-quantized energy states in the ultrafine (1 to 2 nm in diameter) Au NPs built in the conductive filaments in the YSZ films.

## 1. Introduction

Resistive switching (RS) was studied extensively in the last decade due to the prospects of its application in novel nonvolatile computer memory (resistive random access memory, RRAM) [1]. The effect of RS consists in a reversible change of the resistance of a thin dielectric film sandwiched between two conductive electrodes under the external electric voltage [2]. Today's understanding of the RS mechanism in the transition metal oxides is based on a concept of the drift of the oxygen ions O^2−^ (that can be also understood in terms of the drift of the oxygen vacancies, V_O_s) in the external electric field applied between the electrodes [3]. The prevalent mechanism of RS is considered to be the formation and rupture of the nanoscale conductive filaments inside the insulating films consisting of the V_O_s [4].

Recently, a conductance quantization in very thin filaments (with the thickness reduced down to the atomic scale) has been observed experimentally [5, 6]. When a part of the filament (a “bottleneck”) is represented by a chain of V_O_s and a single V_O_ in the chain (or a countable number of these ones) is filled by the O^2−^ ion(s), such a system can be treated as a quantum point contact (QPC) [7–9].

Earlier, we have investigated the RS in the ultrathin (≈5 nm thick) yttria-stabilized zirconia (YSZ) films using conductive atomic force microscopy (CAFM) [10]. This method has been proven to be a powerful tool for studying the RS at the nanometer scale [11, 12], in particular, for imaging and spectroscopy of individual filaments [13]. We have observed the negative differential resistance (NDR) in the current-voltage (*I*-*V*) curves of the contact between the CAFM probe and the individual filaments in the YSZ films. The NDR was related to the resonant electron tunneling through a nanoscale object with a quantized energy spectrum built in the filament. In particular, a cluster of V_O_s in the filament bottleneck separated from the other parts of the filament by the V_O_s filled by the O^2−^ ions could play a role of such a system [14]. This one appears to be an analog of a double-barrier structure (DBS) first considered by Chang et al. [15]. Here, the isolated vacancy cluster plays the role of the quantum well (QW) with the size-quantized energy spectrum and the V_O_s filled by the O^2−^ ions play the role of the tunnel-transparent potential barriers separating the size-quantized system from the rest parts of the filament (the emitter and the collector of the electrons, resp.).

In the present paper, we report on the CAFM investigation of the RS in the YSZ films with embedded single layers of the Au nanoparticles (NPs). The goal of the present work was to confirm experimentally the hypothesis on the origin of NDS in the *I*-*V* curves of individual filaments proposed in the previous study [10] using the Au NPs as the artificial size-quantized systems built in the filaments. We have utilized a well-known fact that the NPs concentrate the electric field inside the dielectric film and, this way, initiate the filament formation at the surfaces at these ones [16]. So far, one can expect the filaments emerging under the CAFM probe potential to grow through the Au NPs. On the other hand, earlier, we have observed NDR in the tunnel spectra of the individual Au NPs in the SiO_2_ [17] and YSZ [18] films on Si substrates originating from the resonant tunneling of electrons between the CAFM probe and the conductive substrate through the size quantized electron energy states in the ultrafine (~1 nm) Au NPs. However, the RS has not been studied in these experiments: the gap voltage *V*_g_ applied between the CAFM probe and the conductive substrate was limited intentionally in order to avoid the RS. In the present study, besides the hysteresis due to the RS, the NDR has been observed in the *I*-*V* curves of individual filaments. The NDR was related to the resonant tunneling of electrons through the size-quantized states in the Au NPs.

## 2. Materials and Methods

The YSZ films with the thickness *d* ≈ 6 nm have been deposited onto the *n*^+^-Si(100) substrates (the specific resistivity of the substrate material was ≈0.005 Ω cm) by high-frequency magnetron sputtering with the use of Torr International® 2G1-1G2-EB4-TH1 vacuum system. The native oxide was not removed from the Si substrates. The deposition was carried out in Ar-O_2_ (50, 50% mol.) gas mixture from a mixed ZrO_2_-Y_2_O_3_ pressed and baked powder target. The molar fraction of Y_2_O_3_ in the target material was ≈0.12. The substrate temperature *T*_g_ was ≈300°C.

The single-layered Au NP arrays embedded into the YSZ films were prepared by the deposition of YSZ/Au/YSZ structures followed by annealing [19]. First, the YSZ sublayers with the thickness *d*_*u*_ = 1 to 3 nm were deposited onto the substrates. Next, the islanded Au layers with the thickness *d*_Au_ ≈ 1 nm were deposited on the YSZ sublayer using direct current magnetron sputtering in the Ar ambient at *T*_g_ ≈ 200°C. Finally, the YSZ cladding layers with the thickness *d*_*c*_ = 5 to 3 nm were deposited in the same conditions, as the YSZ sublayers. The samples were annealed in Ar ambient at 450°C for 1 hour. Some samples have been annealed in the ultrahigh vacuum (UHV) environment (the base pressure was ~10^−10^ Torr) at 300 and 500°C for 1 hour. Also, the ≈6 nm thick YSZ films on the Si(100) substrates without the Au NPs were deposited in the same conditions to serve as the reference samples.

The structure of the YSZ:NP-Au films has been studied by high-resolution cross-sectional transmission electron microscopy (HR X-TEM) by Jeol® JEM-2100/F transmission electron microscope. The accelerating voltage was 180 kV. More detailed information on the procedure of preparation of the YSZ:NP-Au films as well on the results of investigations of the structure and optical properties of the films can be found in [20, 21].

The CAFM measurements were performed in UHV at room temperature using Omicron® UHV AFM/STM LF1 scanning tunneling/atomic force microscope installed into Omicron® MultiProbe™ RM UHV system. The base gas pressure inside the AFM chamber was ∼10^−10^ Torr.

**Figure 1:**
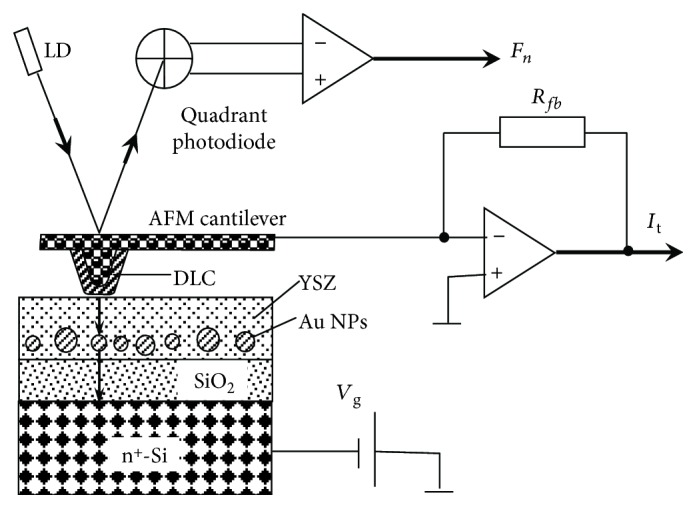
Schematic representation of the experiment on the CAFM investigation of the resistive switching in the YSZ:NP-Au/SiO_2_/*n*^+^-Si(100) films.

The schematic representation of the experiment is shown in Figure 1. NT MDT® NSG-11 DCP™ CAFM probes with diamond-like coating and the tip curvature radius *R*_*p*_ ≈ 70 nm were used. The local electrical conductivity of the YSZ films was investigated by measuring the current between the CAFM probe tip and the sample *I*_t_ subject to the probe position on the investigated sample surface (*x*, *y*) while scanning in the contact mode at fixed bias voltage *V*_g_ between the CAFM probe and the Si substrate. It should be noted that because of the 3 nm thick SiO_2_ film on the Si substrate, the actual voltage drop between the AFM tip and the substrate *V*_g_ drops across a relatively thick structure (9-10 nm in thickness). The relative uncertainty of *I*_t_ measurement in the range 0.1–50 nA was less than 1%. The uncertainty of measurements of *I*_t_ by the STM *I*-*V* converter (preamplifier) built in the AFM/STM was measured by a calibrated resistor with the resistance ≈10 MΩ between the CAFM probe holder and the sample holder (the resistor simulated the tunnel junction between the CAFM probe and the sample surface). The level of intrinsic noise of the STM preamplifier was less than 2 pA. The uncertainty for the *V*_g_ generated by the digital-analog converter of Omicron® SCALA™ electronic control system was ~1 mV.

The RS was studied by recording the cyclic *I*-*V* curves *I*_t_ (*V*_g_) of the probe-to-sample contact. The *V*_g_ was swept in a ramp manner from *V*_min_ to *V*_max_ (*V*_min_ < *V*_RESET_ and *V*_max_ > *V*_SET_) and back from *V*_max_ to *V*_min_ and so forth. Here, *V*_SET_ is the threshold voltage for switching from the “OFF” state to the “ON” one (the “SET” process) and *V*_RESET_ is the one for switching back from the “ON” state to the “OFF” one (the “RESET” process). In another measurement mode, a selected area on the sample surface was scanned in the contact mode with *V*_g_ > *V*_SET_ in order to switch the nanocomposite oxide film within this area from the “OFF” state into “ON” one. In order to switch the film material back from the “ON” state into the “OFF” one, the respective area was scanned again at *V*_g_ < *V*_RESET_. The results of switching were examined by acquiring the CAFM (or current) image *I*_t_(*x*, *y*) at ∣*V*_g_∣ < *V*_SET_, *V*_RESET_.

## 3. Results and Discussion

**Figure 2:**
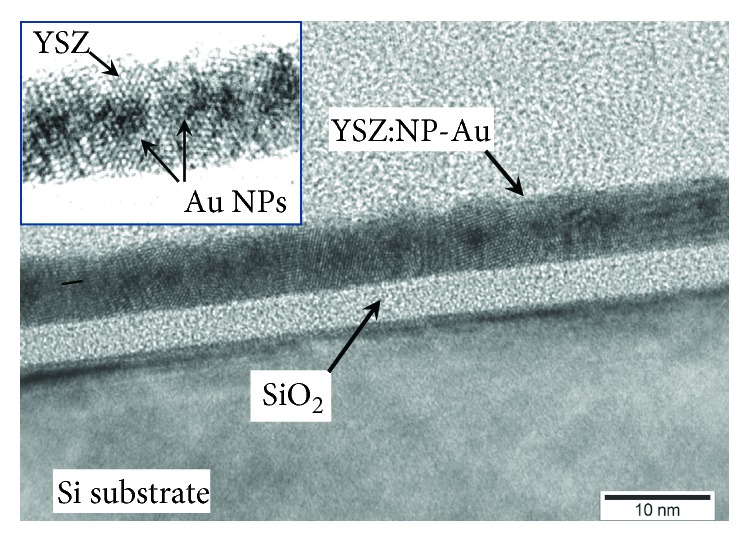
High-resolution X-TEM image of an YSZ (3 nm)/Au (1 nm)/YSZ (3 nm)/SiO_2_/*n*^+^-Si(100) structure annealed in Ar ambient at 450°C for 1 hour.

Figure 2 shows X-TEM image of an YSZ/SiO_2_/Si structure with an Au NP array built in the middle of the YSZ film. According to Figure 2, nearly spherical Au NPs with the diameter *D* = 2.1 ± 0.2 nm were confined in an almost planar layer inside the YSZ film; the thickness of the YSZ film *d* was ≈6 nm. In this sample, the Au NPs have nucleated from an Au film of `≈1 nm in thickness built between the YSZ films of ≈3 nm in thickness each during the annealing process. The density of the NPs *N*_s_ was estimated to be ≈5·10^12^ cm*^−2^*. The mean in-plane spacing between the NP centers was 4.5 ± 0.3 nm.

**Figure 3:**
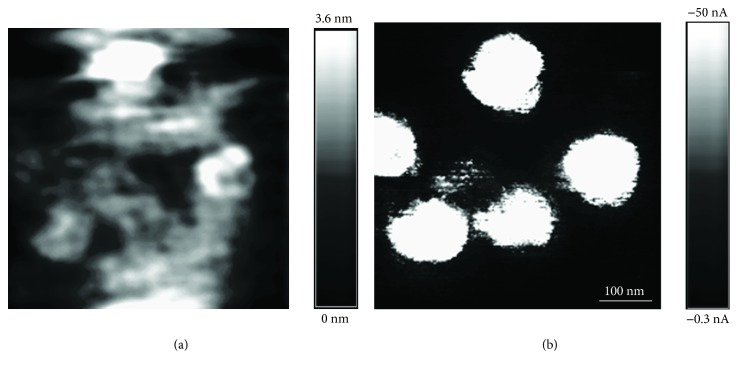
AFM (a) and current (b) images of individual filaments in the YSZ film with the Au NP array (*V*_g_ = −4 V) after the forming by scanning at *V*_g_ = 6 V.

An AFM image of an YSZ/SiO_2_/*n*^+^-Si structure with the Au NPs embedded near the SiO_2_/YSZ interface is shown in Figure 3(a). The nanocomposite YSZ:NP:Au films had rather smooth surfaces. For example, the RMS roughness of the surface of the YSZ:NP-Au film determined from the AFM image shown in Figure 3(a) was ≈0.9 nm.

Figure 3(b) shows a map of the spatial distribution of the probe current *I*_t_ over the YSZ film surface (the current image) measured simultaneously with the AFM one shown in Figure 3(a). Prior to the acquisition of the AFM and current images, a selected area on the YSZ:NP-Au film surface of 500 by 500 nm*^2^* in size was scanned at *V*_g_ = 6 V (i.e., *V*_g_ > *V*_SET_ ≈ 5 V). Note that in Omicron® UHV AFM/STM, *V*_g_ is applied to the sample relative to the probe. The areas of increased CAFM probe current *I*_t_ (the current channels) were observed in the current image of the YSZ:NP-Au film shown in Figure 3(b). These spots were attributed to the electric current flowing through the individual filaments formed in the course of previous scanning. Earlier, we have studied the process of forming the filaments under the electric field between the CAFM probe and the Si substrate [10]. The electric field strength in the YSZ film can be estimated as *F*~*V*_max_/*d*. For *V*_max_ = 5 V and *d* = 5 nm, one gets *F*~10^7^ V/cm. This value is close to the breakdown voltage in YSZ [22] and is well enough to initiate the forming of the filaments.

As it has been shown in [10], the filaments grow preferentially in the places on the sample surface, where some defects are located inside the YSZ film, such as point defects (or defect clusters) in YSZ and some hillocks or protrusions on the substrate surface. These defects concentrate the electric field between the CAFM probe and the substrate promoting the filament growth. In the case of the YSZ:NP-Au films investigated in the present study, most likely, the Au NPs were the defects promoting the filament growth. As it has been already mentioned above, the metal NPs inside the dielectric films are known to improve the RS playing a role of the electric field concentrators [16].

The sizes of the current channels (~100 nm) were of the same order of magnitude as the values of *R*_p_ (≈70 nm). Earlier, we have shown the sizes of the current images of the localized electron states inside the tunnel transparent dielectric films (e.g., the metal NPs, point detects, and defect clusters) to be determined by the size and shape of the probe-to-sample contact area and not to depend on the defect size itself [23]. This is a manifestation of the convolution effect [24] which, in turn, is a particular case of the general principle of the theory of measurements claiming the result of any measurement to be a convolution of the object function with the apparatus one.

(1) $$\begin{array}{c} I_{t}\left(x,y\right)=\int_{-\infty }^{\infty }\int_{-\infty }^{\infty }K\left(x-x^{\prime },y-y^{\prime }\right)f\left(x^{\prime },y^{\prime }\right)dx^{\prime }dy^{\prime }. \end{array}$$

In the case of imaging the filaments, the kernel *K*(*x*, *y*) in (1) represents the area of the tip-to-sample contact. The size of the contact area *D*_p_, which can be estimated from the solution of Hertz problem [25], is much greater than the one of the filament tip at the film surface (which, in turn, can be as low as the size of a single V_O_ in the ultimate limit). So far, the objective function of the filament *f*(*x*^`^, *y*^`^) can be approximated by Dirac delta function *δ*(*x*^`^, *y*^`^). Thus, according to (1), *I*_t_(*x*, *y*) → *K*(*x*, *y*). In other words, when imaging the filaments by an AFM probe with relatively large *D*_p_, one rather gets the image of the tip-to-sample contact imaged by a *δ*-function-like filament.

**Figure 4:**
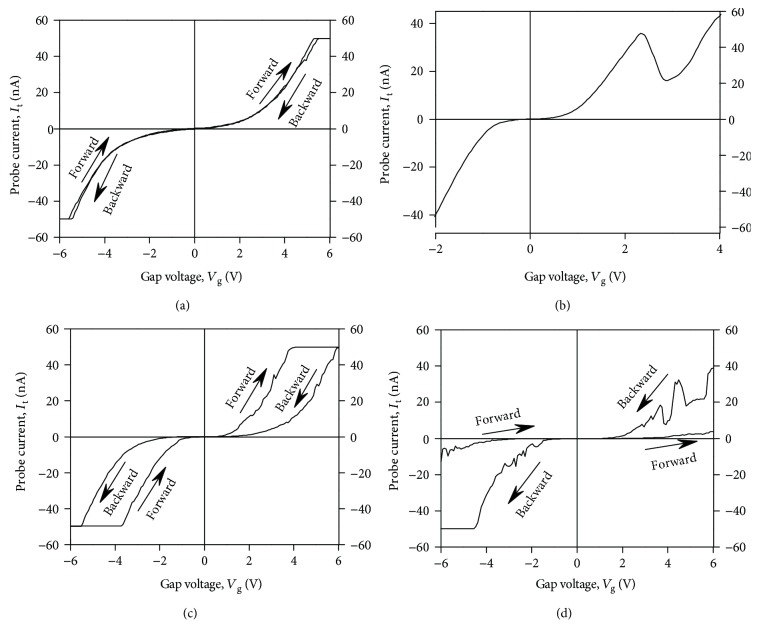
Typical *I*-*V* curves of the contact of CAFM probe to the YSZ:NP-Au/SiO_2_/*n*^+^-Si(100) film measured after forming by scanning at *V*_g_ = 6 V: (a) an *I*-*V* curve typical for the MOS structures with tunnel transparent dielectrics; (b) an *I*-*V* curve with NDR; (c) a cyclic *I*-*V* curve with the bipolar-type hysteresis; (d) a cyclic *I*-*V* curve with the hysteresis and NDR. The curves (c) and (d) were measured on the individual filaments.

Figure 4 presents typical *I*-*V* curves of the contact of CAFM probe to the YSZ:NP-Au film observed in the experiment. These *I*-*V* curves can be classified into the following four categories.

- The monotonous *I*-*V* curves (Figure 4(a)) typical for the metal-oxide-semiconductor (MOS) structures with tunnel transparent dielectric [26].
- The *I*-*V* curves with NDR (Figure 4(b)): the curves of this type have been observed earlier [17, 18] in the tunnel-transparent dielectric films with embedded Au NPs and were attributed to the resonant tunneling between the CAFM probe and conductive Si substrate through the size-quantized states in the ultrafine Au NPs (~1 nm in size).
- The *I*-*V* curves with the butterfly-type hysteresis loops (Figure 4(c)): the *I*-*V* curves of this kind have been reported in the literature many times (see, e.g., [27, 28]) and were attributed usually to the bipolar RS in the dielectric films under the electric field between the CAFM probe and the conductive substrate.
- The *I*-*V* curves with a pronounced hysteresis and with NDR at the same time (Figure 4(d)).

It should be stressed here that the principal difference between the cases (i) and (ii) on one hand (Figures 4(a) and 4(b), resp.) and the cases (iii) and (iv) on the other hand (Figures 4(c) and 4(d), resp.) consists in the following. In the former two cases, there were no hysteresis loops in the *I*-*V* curves whereas in the latter two ones the *I*-*V* curves were featured by a pronounced hysteresis. These cases differ from each other by the ramp voltage sweep range. The limits of *V*_g_ sweep in the cases (i) and (ii) were set less than *V*_SET_, *V*_RESET_ ≈ 5 V, intentionally in order to avoid the RS. In contrary, in the cases (iii) and (iv), the ramp voltage limits were set greater than *V*_SET_, *V*_RESET_, that resulted in the manifestation of the hysteresis loops in the cyclic *I*-*V* curves due to the RS.

It is worth noting also that there are many reports in the literature on the investigations of RS by CAFM in ambient air (see, e.g., [12, 28] and references therein). We have also tried to reproduce the measurement of RS in the YSZ/Si films by CAFM in air using NT-MDT® Solver Pro™ AFM. We have observed the *I*-*V* curves of the tip-to-sample contact with the hysteresis loops similar to the ones presented in Figure 4(c). However, the stability of the *I*-*V* curves measured in air was much poorer than of the ones measured in UHV. We attributed this fact to the impact of the humidity contamination in ambient air: the YSZ films absorb the oxygen ions from the contamination layer that reduces the concentration of the oxygen vacancies in the YSZ films and, this way, suppress the resistive switching.

**Figure 5:**
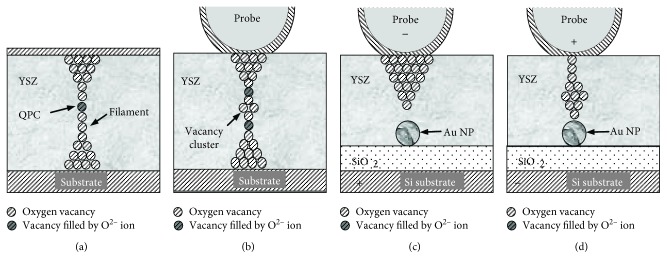
The schematic representation of the conductive filaments inside an YSZ film: (a) a single V_O_ in the filament “bottleneck” filled by O^2−^ ion [7]; (b) a vacancy cluster separated from the rest parts of the filament by the V_O_s filled by O^2−^ ions [10]; (c, d) an Au NP built in the filament “bottleneck.”

The cyclic *I*-*V* curves of the kind (iv) similar to the ones presented in Figure 4(d) have been observed earlier on the YSZ/Si films without the NPs [10] and were ascribed to the resonant tunneling of electrons through some quantum objects with a discrete energy spectrum embedded into the filaments. This suggestion was based on further development of the concept of a QPC in the filament “bottleneck” (Figure 5(a)) proposed earlier in [7, 9]. Namely, if two V_O_s in a vacancy chain (representing the bottleneck in the filament, Figure 5(b)) filled by the O^2−^ ions separate a vacancy cluster from other parts of the filament, such a system can manifest NDR in the *I*-*V* curves because of resonant electron tunneling through the discrete energy states in the vacancy cluster. Such systems could form in the course of fast dissolving of the filaments during the RESET process in strongly nonequilibrium conditions [14].

**Figure 6:**
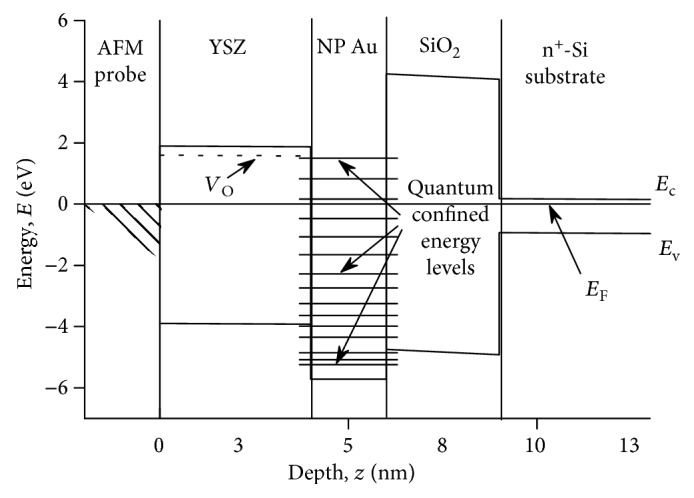
Calculated equilibrium one-dimensional band picture (300 K) across an Au NP with the diameter *D* = 2 nm embedded into the YSZ film of ≈6 nm in thickness on the *n*^+^-Si(100) substrate with native oxide.

In the present study, we attribute the simultaneous observation of the NDR and the hysteresis in the *I*-*V* curves of the contact of CAFM tip to the YSZ:NP-Au films (Figure 4(d)) by resonant electron tunneling through the size-quantized states in the nanometer-sized Au NPs. The filaments growing through the NPs play a role of the emitters (or collectors) of the electrons.  Figure 5(c) presents a schematic representation of this suggested mechanism of NDR whereas Figure 6 presents an equilibrium one-dimensional band picture of the CAFM tip contact with the diamond-like coating and the YSZ:NP-Au/SiO_2_/*n*^+^-Si(100) film across an Au NP with the diameter *D* = 2 nm embedded into the YSZ film with *d* ≈ 6 nm near the YSZ/SiO_2_ interface (i.e., *d*_*u*_ = 1 nm, *d*_*c*_ = 5 nm). The band picture was calculated just for the layer thicknesses as in the structure investigated in the present study extracted from the X-TEM results (Figure 2). The calculation procedure details were published in [29]. The heights of the energy barriers in the YSZ/Au/YSZ/SiO_2_/*n*^+^-Si(100) structure were obtained from the results of the ballistic electron emission microscopy/spectroscopy (BEEM/BEES) measurements [29, 30]. It is obvious from Figure 6 that the contact of CAFM tip to the YSZ/Au/YSZ/SiO_2_/Si structure is an asymmetric DBS, where the SiO_2_ film plays the role of the first tunnel barrier. As for the second barrier, it is known that for the operation of a memristive cell, it is not necessary for the filament to shortcut the electrodes completely in the “ON” state. If a thin (tunnel-transparent) insulating layer remains between the filament tip and the counter-electrode, the tunneling of electrons through the thin insulating layer appears often to be essential to provide enough conductivity of the whole memristive cell in the “ON” state [31]. So far, if a thin insulating layer (where all V_O_s are filled by the O^2−^ ions) remains between the filament tip and the Au NP, this insulating layer could play the role of the second tunnel barrier of the DBS.

Also, the size-quantized electron energy levels in Au NP calculated according to the model of a spherical quantum dot (QD) with finite potential barrier height [32] for an Au NP with *D* = 2 nm in the YSZ matrix are shown in Figure 6. The details of calculations were given in [29] as well. The mean energy gap between the size-quantized energy levels near Fermi energy in Au NP with *D* = 2 nm can be estimated to be ≈0.6 eV. Earlier, BEEM/BEES was applied for the investigation of the quantum size energy spectrum in Au NPs built in YSZ/Si structures deposited in the same conditions as the ones utilized to prepare the YSZ:NP-Au/SiO_2_/*n*^+^-Si films investigated in the present study [29]. The energies of the size quantization states in the Au NPs measured experimentally were consistent with the ones calculated according to the model of spherical QD. Thus, the conditions for the resonant electron tunneling were readily present in the investigated YSZ:NP-Au films.

**Figure 7:**
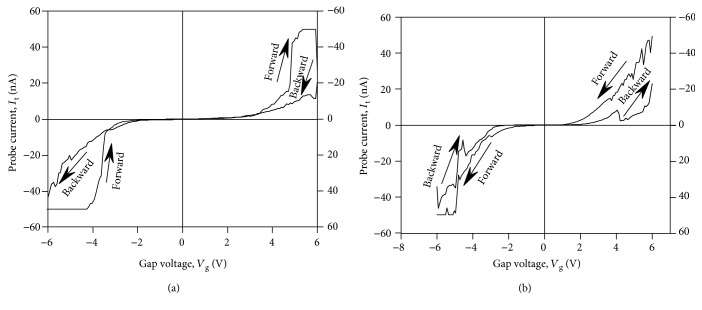
Typical *I*-*V* curves of contact of CAFM tip to the YSZ:NP-Au/SiO_2_/*n*^+^-Si(100) film measured on individual filaments after forming by scanning at *V*_g_ = 6 V (a) and −6 V (b).

In the model described above, the filaments are suggested to grow from the CAFM tip to the Au NP (i.e., the forming should be performed at *V*_g_ > 0) and the injection of the electrons from the filament into the NP takes place at *V*_g_ > 0 as well. This is just the case of the cyclic *I*-*V* curve presented in Figure 4(d). It is worth noting that one can perform the forming either from the CAFM tip (Figure 5(c)) or from the Au NP (Figure 5(d)). The direction of the filament growth is determined by the polarity of *V*_g_ when performing the forming process by scanning. Correspondingly, the polarity of the SET and RESET processes (i.e., the directions of the hysteresis loops in the cyclic *I*-*V* curves) is different in the two above cases (cf. Figures 7(a) and 7(b)).

It is worth noting that the hysteresis in the *I*-*V* curves could potentially originate also from the charging of the Au NPs by the electrons injected into these NPs either from AFM tip or from Si substrate [33] or from the ion migration polarization of the YSZ film [34]. However, the hysteresis in the *I*-*V* curves has been observed not only in the YZS films with embedded Au NPs but also in the YSZ films without these ones as well (see, e.g., [10]). Therefore, the hysteresis in the *I*-*V* curves of the YSZ:NP-Au films should be attributed rather to the RS than to the charging of NPs.

On the other hand, the hysteresis in the *I*-*V* curves of the metal-insulator-metal (MIM) stacks due to the ion migration polarization is featured by the nonzero reverse current at zero bias voltage (the discharge current). Note that both branches of the cyclic *I*-*V* curve (the forward branch as well as the reverse one) cross the point *V*_g_ = 0, *I*_t_ = 0 (see Figure 4(c)). This is typical for the RS and is not intrinsic to the ion migration polarization.

**Figure 8:**
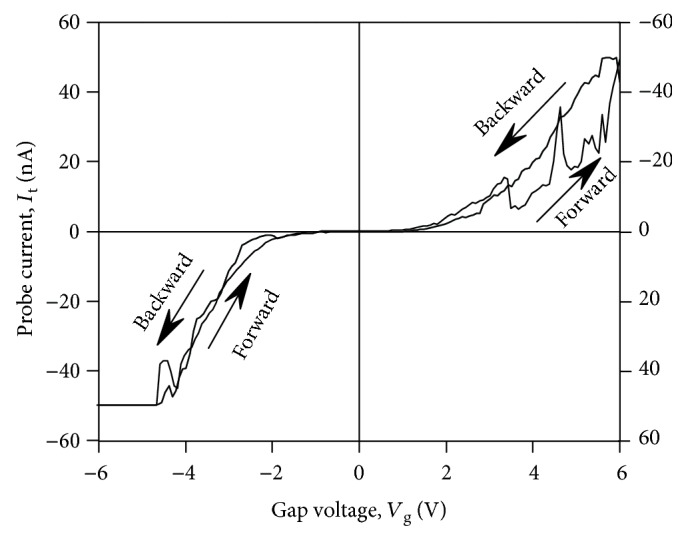
An example cyclic *I*-*V* curve of the CAFM probe contact to an YSZ:NP-Au/SiO_2_/*n*^+^-Si film with a dynamic SET process.

Also, when analyzing the cyclic *I*-*V* curves with hysteresis, one should distinguish between the case of the *I*-*V* curves with NDR (like the ones shown in Figure 4(d)) and the case when the *I*-*V* curves contain some kinks or bumps during the SET process. This effect has been reported in the literature extensively and is usually referred to as the “dynamic SET process” (see, e.g., [35, 36]). The dynamic SET process in the memristive cells with the macroscopic (micrometer-sized) electrodes was usually attributed to the growing and burning out of the filaments due to Joule overheating because of too large electric current flowing though these ones. As a result, the new filaments are being involved into the conductivity between the metal electrodes instead of the burned ones [37]. So far, the SET process in this case could involve several “failed attempts.” As for the CAFM measurements, we have also observed such a “dynamic” RS of the dielectric films under the CAFM probe (see, e.g., Figure 8). In the case of CAFM investigations, more than one filament could hardly occur under the CAFM probe contact to the dielectric film. So far, in this case, such a dynamic RS could be attributed to burning out and restoring a single filament. According to [14], such a process is possible in the strongly nonequilibrium conditions. The key feature allowing distinguishing between the “dynamic” RS and the manifestation of NDR due to the resonant tunneling is the fact that the former phenomenon is usually observed during the SET process (as in the *I*-*V* curve shown in Figure 8) whereas the latter can be manifested in the backward branches of the *I*-*V* curves in the “ON” state like in the *I*-*V* curve shown in Figure 4(d).

## 4. Conclusion

In the present study, we have investigated the local resistive switching in the ultrathin yttria-stabilized zirconia films with embedded Au nanoparticles using conductive atomic force microscopy to measure the electric current through individual filaments. Besides the butterfly-type hysteresis loops in the *I*-*V* curves of the contact between the CAFM tip and the sample typical for the bipolar resistive switching in the metal oxides, the negative differential resistance has been observed. The effect was related to the resonant tunneling through the size-quantized electron energy states in Au nanoparticles of ~1 nm in size built in the filaments.

## Data Availability

All data supporting the published results are included in the manuscript.
